# Complex Processes from Dynamical Architectures with Time-Scale Hierarchy

**DOI:** 10.1371/journal.pone.0016589

**Published:** 2011-02-10

**Authors:** Dionysios Perdikis, Raoul Huys, Viktor Jirsa

**Affiliations:** Theoretical Neuroscience Group, UMR6233 Institut Science du Mouvement, University of the Mediterranean, Marseille, France; Mount Sinai School of Medicine, United States of America

## Abstract

The idea that complex motor, perceptual, and cognitive behaviors are composed of smaller units, which are somehow brought into a meaningful relation, permeates the biological and life sciences. However, no principled framework defining the constituent elementary processes has been developed to this date. Consequently, functional configurations (or architectures) relating elementary processes and external influences are mostly piecemeal formulations suitable to particular instances only. Here, we develop a general dynamical framework for distinct functional architectures characterized by the time-scale separation of their constituents and evaluate their efficiency. Thereto, we build on the (phase) flow of a system, which prescribes the temporal evolution of its state variables. The phase flow topology allows for the unambiguous classification of qualitatively distinct processes, which we consider to represent the functional units or modes within the dynamical architecture. Using the example of a composite movement we illustrate how different architectures can be characterized by their degree of time scale separation between the internal elements of the architecture (i.e. the functional modes) and external interventions. We reveal a tradeoff of the interactions between internal and external influences, which offers a theoretical justification for the efficient composition of complex processes out of non-trivial elementary processes or functional modes.

## Introduction

The notion that basic elementary units serve as constituent building blocks (or primitives) for the composition of complex functional processes and behaviors, be they motoric, perceptual or cognitive, is widely adhered to in the biological and life sciences. For instance, the vocal behavior of singing birds comprises functional elements with distinct associated time scales, such as notes and syllables (groups of notes), that are represented hierarchically in the avian forebrain [1]. The time-scale separation that is inherent to the production of bird song has recently been proposed to also underlie its perception [2]. Similar hierarchical decompositions are likely to be involved in (human) speech perception [3], where phonemes are known to constitute meaningful categories relevant for communication [4]. In a similar spirit, precise manual movements, as evident in handwriting, may result from the dual activity of a sequential controller interacting with a trajectory generator [5]. Finally, perception-action architectures in artificial intelligence may initiate learning cycles using primitives to acquire complex skills [6].

These examples readily indicate that the literature is replete with functional architectures with a hierarchy of processes operating on different time scales. These architectures often contain a considerable degree of detail and specificity, in order to account for the specific features of a particular application. However, it also limits their generality. A noticeable exception in that regard is the approach developed over the last years by Friston and colleagues [7]. The main premise of their approach is that the brain's structural and functional organization mimics the causal structure in the environment through the free-energy principle. The framework is nicely illustrated in [2], where environmental dynamics are inherently structured as a temporal hierarchy. The authors exemplified their approach in the context of bird song, generated as a two-leveled coupled Lorenz attractor operating on distinct time scales. Perceptually, the slower evolving causal dynamics (singing bird) are retrieved via a Bayesian inversion of the generative model from the temporal structure in the fast environmental changes (sound waves). Powerful and promissory as the approach is, the question as to what identifies and classifies the elementary units of which complex behaviors are constructed remains unaddressed.

A variety of attempts, nevertheless, to identify elementary units has been pursued, mainly so, to our best knowledge, in the context of motor control. There, a pertinent question is how the nervous system reliably controls, stores, and activates complex motor behavior in light of changing environmental context and neuro-muscular system's complexity [8], [9]. Elementary functional units in movement sciences are referred to as motor primitives or synergies [9]–[15] in different domains stressing distinct albeit associated aspects of motor control. In that regard, a synergy refers to a temporal and functional organization in terms of “a group of muscles often spanning a number of joints that is constraint to act as a single functional unit” [16]. In contrast, a motor primitive (originally) refers to mechanical consequences (force fields) that are the resultant of the stimulation of hard-wired neural circuitry (see below) although it has recently also been used in a broader sense designating a ‘functional motor control unit’. Founded in the cognitive information-processing perspective, Schmidt [9], [17] proposed that action is controlled on the basis of a limited number of functional modules, referred to as generalized motor programs (GMPs). Accordingly, each GMP contains fixed ‘algorithms’ to control a particular class of actions (e.g., overhand throwing) such that it assures the class-invariant features (presumably, the order of events, relative timing, and relative force). Whenever a GMP is called upon several adjustable parameters (time duration, force, and effectors used) are specified so as to satisfy the context-specific task constraints. An altogether different perspective stems from Mussa-Ivaldi and colleagues, namely that neural circuits in the spinal cord are organized in terms of functionally distinct modules. Experimental studies on spinalized frogs revealed that stimulation of a particular spinal cord circuit evoked reproducible contractions in groups of muscles, inducing module-specific force fields—the motor primitives [18], [19]. The simultaneous activation of multiple modules leads to the vectorial superposition of the corresponding force fields and as such may generate a large variety of motor behaviors [10], [20]. Others, still, have claimed that (stable) fixed points and limit cycles – dynamical structures that are associated with discrete and rhythmic movements, respectively – constitute the fundamental building blocks that are at the nervous system's disposal to compose actions [14], [21], [22]. Importantly, their attractiveness guarantees the units' functionality in the face of perturbations, which (among others) motivates their utility in humanoid robotics design [23], [24]. Also, turning “on” or “off” dynamical systems out of an available “alphabet”, depending on different behavioral situations, is the basis of one popular control strategy in the robotics and hybrid automatic control literature, known as Motion Description Languages (MDLs) [25]–[27]. Outside of the motor control domain, the notion of primitives is debated in the context of visual perception, where Marr's [28] proposal that local geometrical properties serve as visual primitives has dominated the debate for a long time. Recently, however, evidence was found that the topologies of static [29], [30] as well as dynamic [31] visual scenes count as primitives underlying pattern recognition (see [32] and [33] for contrasting views).

This brief overview readily indicates that the various approaches stress different aspects adhering to motor primitives, namely class-defining invariance and within class variation, executive stability (i.e., maintenance of performance in the presence of perturbations), and assemblability (i.e., the notion that primitives can be assembled and embedded into a larger functional organization). No single approach, however, incorporates all three features. Below, we outline a general dynamical framework for functional two-layered architectures for the production of complex behavioral processes incorporating all three requirements. These architectures contain two ingredients: functional modes that are defined in terms of phase flows, which define the evolution of the state variables in their state space; and signal operating upon the phase flows. These operational signals may act on different time scales than the functional modes. We define four representative functional architectures based on the notion of time scale separation and evaluate their efficiency. The latter is achieved by calculating the ‘informational content’ of the operational signals and the ‘complexity’ of the functional modes (see the *Measures of Complexity* section for the formal implementation of these intuitive notions). Please notice that we do not refer to the functional elementary entities out of which complex processes can be composed as primitives or synergies as these concepts have particular connotations in the motor control literature (see above) and the framework here outlined is not limited to motor behavior.

**Figure 1:**
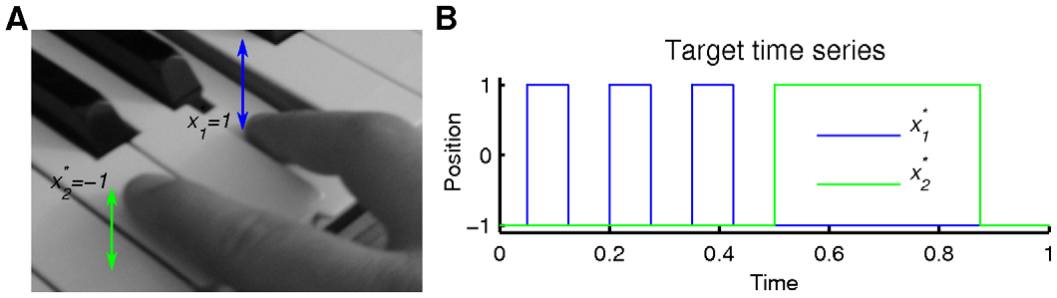
Illustration of our toy example. One finger (blue) plays three notes in sequence followed by a fourth note of a longer duration played by another finger (green). The fingers' positions (*x*_1_*, *x*_2_*) are displayed in the right panel as a function of time.

We illustrate our framework in the context of the control of a sequential movement, mimicking the well-known four introductory notes of Beethoven's 5^th^ symphony, as a toy example. We portray the execution of this musical phrase as the sequence of three piano key presses of equal duration followed by a forth one of longer duration (Figure 1). Each key press is realized by a different finger. Constructing a hierarchy of processes modeling the entire sequence we evaluate the functional architecture with regard to its mathematical form rather than from the specific instantiation of the composite processes. Our perspective though should not be limited to motor behavior only. Quite on the contrary: we propose that phase flows constitute a generic language of the nervous system for the coding of cognitive (in the broadest sense) phenomena, although this is presently a conjecture.

## Methods

### Phase flows and control

**Figure 2:**
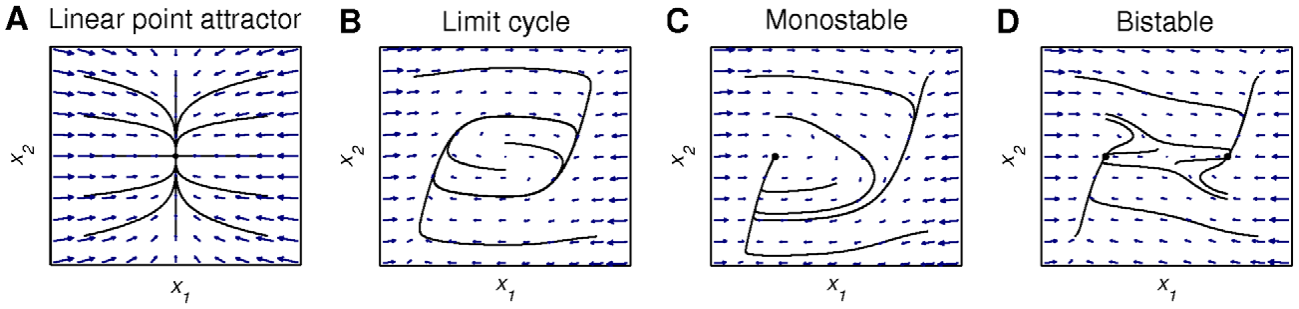
Examples of functional modes. Phase flows corresponding to a linear point attractor, a limit cycle, a monostable, and a bistable condition in panels A, B, C, and D, respectively (see also equations (3), (4) and (2) ignoring operational signal *σ*(*t*) where present). Blue arrows sketch the vector fields of the flows in phase space, here spanned by position *x*_1_ and velocity *x*_2_, and describe the system's evolution as a function of its state (*x*_1_, *x*_2_). Black lines represent trajectories (i.e., realized system evolutions) for various initial conditions; black circles represent stable fixed points (i.e., points where 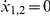). The black closed orbit (panel B) represents a stable limit cycle (a circular structure describing oscillatory phenomena). A separatrix (i.e., a structure that locally separate flow with opposing directions) exists in the monostable and bistable condition (panel C and D), and can particularly well be gleaned from panel D, where two trajectories with initial conditions close to each other approximately in the middle of the phase space, diverge into different directions. Fixed points, limit cycles, and separatrices are so-called topological structures.

Dynamical systems are either autonomous or non-autonomous, so defined via the absence versus presence of an explicit time-dependent component, respectively. Autonomous, deterministic, and time continuous systems are unambiguously described by the flow in phase (or state) space, which provides a quantitative description of a system's evolution as a function of its current state (see Figure 2). The phase flow topology uniquely determines a system's qualitative behavior. Another way of putting this is that phase flow topologies conserve a system's dynamic invariant features—thus identifying all behavioral possibilities within a class in a model-independent manner. While a system's flow completely describes an autonomous (deterministic) system's behavior, the behavior of non-autonomous systems additionally depends on some (external) time-dependent influences. In its most general formulation, we can describe a functional architecture through its phase flow as
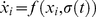
(1)where *x_i_* for *i* = 1…*N* are the system's state variables (the dot indicates the time derivative) and *σ*(*t*) represents a time-dependent influence – the operational signal – that, if constant in time, renders the process autonomous. A functional mode is defined through equation (1) where 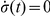 for all *t* and the dynamic repertoire is the set of functional modes. To anticipate, intuitively it makes sense to assume that the costliness associated with a process is a function of the presence versus absence of an operational signal, and furthermore depends on the complexity of the functional mode and the operational signal (if present). Different functions *f* may preserve particular invariant properties while allowing for variation in detailed trajectory variability as imposed, for instance, by particular task constraints, which is easily illustrated in the context of limit cycles. Limit cycles may contain various damping and stiffness terms (e.g., van der Pol, Rayleigh, Duffing, and others, [34]) that all are closed orbits in phase space. This formulation embraces the characteristics mentioned above: class-defining invariance and within class variation, executive stability, and assemblability.

**Figure 3:**
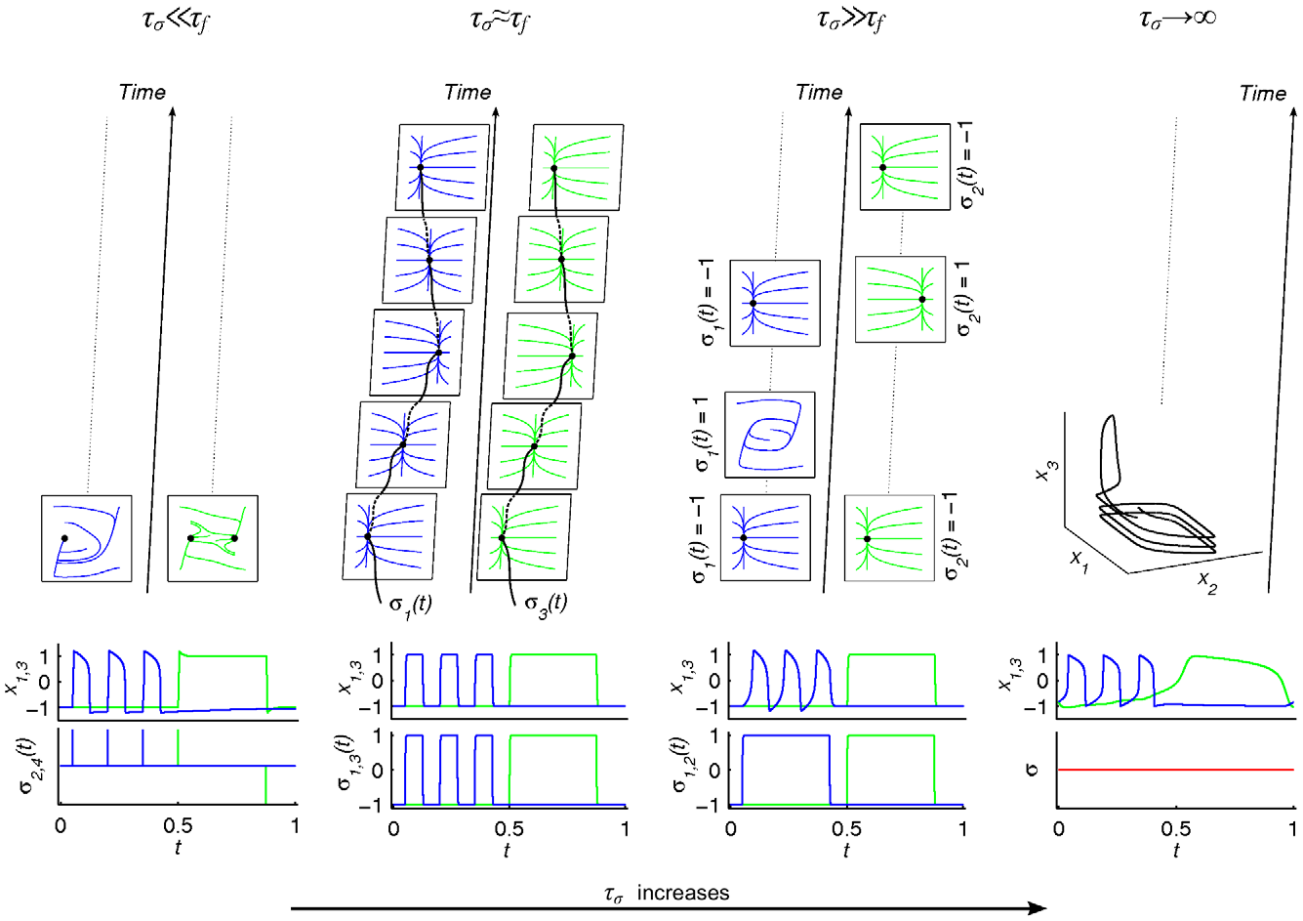
Overview of the four functional architectures. Each column represents a functional architecture with time scale separation as indicated at the top. Lower row: Time series depicting the operational signals *σ_i,j_*(*t*) (lower graph; *i*, *j* index the system's dimensions –in columns 1 and 2- or fingers –in column 3- where *σ* operates upon) and the system's output *x*_1,3_(*t*) (upper graph; state variables accounting for position). Blue and green lines represent *σ_i_* and *x*_1_ versus *σ_j_* and *x*_3_, respectively. Upper rows: The time evolution is indicated by the arrows. Each square panel in the upper rows represents the phase space of a particular functional mode; sequential panels (in time) indicate changes in the functional modes; dotted lines indicate the persistence of a particular mode (until substituted by another one). Paired panels (left, right) represent the modes corresponding to finger 1 and 2, respectively (except for the fourth column where the two fingers are coupled and where only three out of the four dimensions of the system's phase space can be shown). From left to right; first column (*τ_σ_*≪*τ_f_*): *σ*_2,4_ provide instantaneous functional kicks to the modes (see equation (2)); second column (*τ_σ_*≈*τ_f_*): fixed points are driven by *σ*_1,3_ through phase space (one movement cycle depicted only –also see equation (3)); third column (*τ_σ_*≫*τ_f_*): *σ*_1,2_ sequentially select distinct functional modes (see equation (5)); fourth column (*τ_σ_*→∞): *σ* = constant (has no effect), i.e., the system is entirely autonomous. Notice that the more the time scales of the operational signals and the functional modes differ, the more the role of the operational signals decreases and the complexity of the phase flows involved increases.

The operational signal *σ*(*t*) operates (upon) the functional modes and generally will not be independent of *x_i_*. Here we wish to focus on the causal effects of the operational signal upon the functional modes. Let *τ_f_* and *τ_σ_* denote the time scales corresponding to a particular functional mode and operational signal *σ*(*t*) respectively. For different functional architectures, *τ_σ_* may operate on various time scales relative to *τ_f_* and could in principle span a continuum of scales. Here, we choose four different instantiations of time scale separations (see Figure 3 for an overview). In cases in which *σ*(*t*) acts much faster than the functional mode (i.e., *τ_σ_*≪*τ_f_*), *σ*(*t*) operates upon the mode (exemplified below as *Scenario 1*). In those cases where *σ*(*t*) acts on a time scale similar to that of the functional mode (i.e., *τ_σ_*≈*τ_f_−Scenario 2*), *σ*(*t*) may be said to operate the functional mode. In *Scenario 3* we consider the case where *σ*(*t*) acts much slower than the functional mode (*τ_σ_*≪*τ_f_*). Finally, in the fourth architecture *σ*(*t*) can be considered as time-independent (i.e., *σ*(*t*)≈ constant during the functional process or equivalently *τ_σ_*→∞*−Scenario 4*). All scenarios are exemplified below.

### Functional hierarchies exemplified

We illustrate our approach by computationally implementing the execution of our toy example (Figure 1) using qualitatively distinct functional modes (phase flows) in the four different scenarios. While each specific model implementation exemplifies one of the four scenarios, it is important to notice that they (merely) serve as placeholders representing phase flows: in each case numerous other phase flows could be implemented, but the scenarios are set apart via their corresponding time scale separations. We have computationally implemented various other realizations of individual phase flows (not shown here), and all other results remained the same. The functional modes used in the scenarios below can be conceived of as ‘minimal’ implementations such that they avoid capacities that are irrelevant for the ‘task’ at hand (i.e., they are not functionally redundant). The significance of the role of the functional mode(s) in the scenarios depends, by large, on the degree of time scale separation. The delineation of (invariant) functional modes and (varying) operational signals *σ*(*t*), however, allows for the quantification of the (operational) influence required for a given (complex) behavioral process as well as the functional mode's complexity (see below).

**Figure 4:**
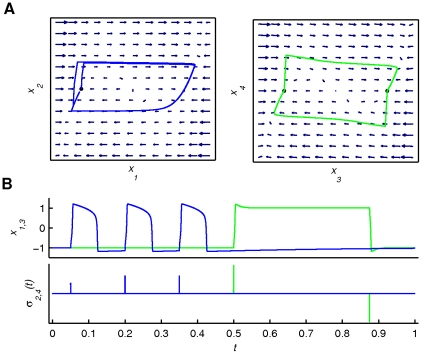
Illustration of *Scenario 1*. *Scenario 1* (see equation (2)) shows the vector fields of the phase flows (monostable and bistable) together with the output trajectories (panel A) and the output time series (positions *x*_1,3_ and operational signals *σ*_2,4_(*t*) -panel B). Blue and green discriminate between first and second finger; a small black filled circle denotes an attracting fixed point. The phase flows remain constant during the functional process (*τ_σ_*≪*τ_f_*), while the amplitude of the operational “kicks” has been regulated in order to optimize the output (in any case maintaining the characteristics of a *δ*-function like stimulus with very large amplitude and minimal duration). Note that *σ*(*t*) operates upon the second and fourth dimensions of *x* that account for the velocities of the fingers' movements.

**Figure 5:**
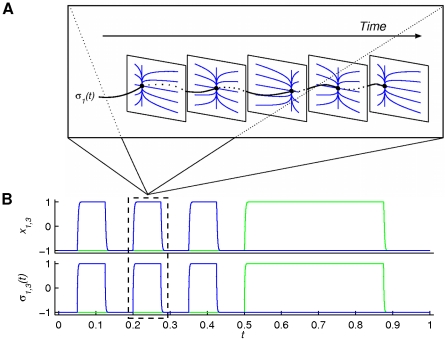
Illustration of *Scenario 2*. *Scenario 2* (see equation (3)) shows a sketch of the phase flows (linear point attractor -panel A) as well as the output time series (positions *x*_1,3_ and operational signals *σ*_1,3_(*t*) -panel B). Colour coding and fixed point notation are the same as in the previous figure. A single pulse of *σ*_1_(*t*) and its effect on the phase flow of the first finger are blown up in panel A, depicting five characteristic instances of the phase flow. The phase flows change at the same time scale as the functional process (*τ_σ_*≈*τ_f_*), since the position of the attracting equilibrium point is constantly assigned by the operational signal.

**Figure 6:**
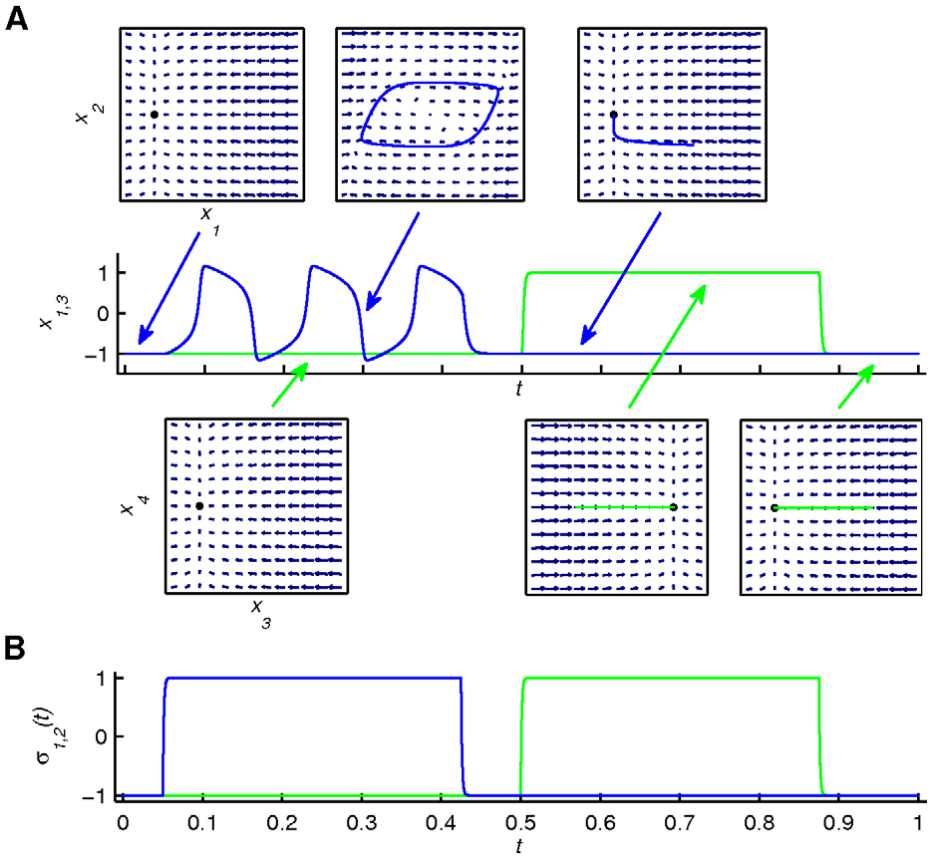
Illustration of *Scenario 3*. *Scenario 3* (see equation (5)) shows the temporal succession of the phase flows (linear fixed points and limit cycle) together with the corresponding concurrent segments of the output trajectories (panel A) as well as the output time series (positions *x*_1,3_ in panel A and operational signals *σ*_1,2_(*t*) in panel B). Colour coding and fixed point notation are the same as in the previous figures. The arrows are pointing at segments of the output time series during which a phase flow is activated (and thus dominates the output dynamics). The actual moment and duration of activation of each phase flow can be directly inferred by the operational signal plot in panel B. The phase flows change only at critical moments during the functional process due to the slow change of *σ*(*t*). Note that *σ*_1_(*t*) and *σ*_2_(*t*) operate upon the first and second finger phase flows respectively.

**Figure 7:**
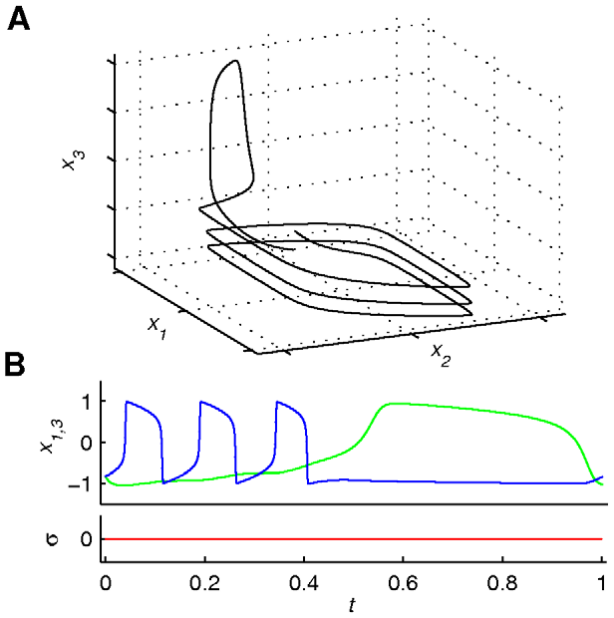
Illustration of *Scenario 4*. Scenario 4 (see equation (6)) shows the phase flow (3-dimensional projection) through the output trajectory (panel A) as well as the output time series (positions *x*_1,3_ and operational signal *σ*(*t*) –panel B). Blue and green discriminate between first and second finger (coupled) only for the time series plot. The phase flow remains constant during the functional process since there is no operational signal involved. Although this is just a 3-dimensional projection of the phase flow, one can observe the spiral of the three movement cycles of the first finger on the plane *x*_1_−*x*_2_, followed by one more on what would be the plane *x*_3_−*x*_4_.

The functional modes implemented below consist of 4-dimensional phase flows (two dimensions per effector). In all cases, (*x*_1_, *x*_2_, *x*_3_, *x*_4_), are the state variables of the system, *T*_1,2_ are the effectors' main time constants while *k*_1,2_ introduce a time scale separation between the state variables of each effector's phase flow. Thus, (*x*_1_, *x*_2_), *T*_1_, *k*_1_ and (*x*_3_, *x*_4_), *T*_2_, *k*_2_ refer are associated with the first and second effector respectively. At the same time, the state variables (*x*_1_, *x*_3_) correspond to the effectors' positions, while (*x*_2_, *x*_4_) correspond to their velocities. This notation is used in Figures 4, 5, 6 and 7 describing the results and in the presentation of *Scenario 4* below. The same notation is used for the operational signals where the indexes of *σ* correspond to either the equation's state variables (1 to 4 in *Scenarios 1* and *2*) or to an effector (1 or 2, in Scenario 3). For reasons of brevity, we only present the two-dimensional phase flows used to model either both or each one of the effectors for *Scenarios 1–3* below, since the two effectors are modeled as uncoupled (and can thus be presented separately). The 4-dimensional system in *Scenario 4* is presented entirely as its corresponding two effectors are coupled. No claim for the generating mechanisms of the operational signals is made in the present work. The ones used in the simulations where chosen such as that the resulting multidimensional operational signals are non-autonomous and their different dimensions are uncorrelated. All simulations were carried out in MATLAB, while a Runge-Kutta algorithm of 4^th^ order has been used for the integration of the dynamical systems. Further details on the models and simulations can be found in the Supporting Information (Text S1).

#### Scenario 1

In architectures where *τ_σ_*≪*τ_f_*, the phase flows maintain a constant structure, since *σ*(*t*) operates only instantly on them. The phase flow may account for more than one sub-function coded in the phase space (in cases of multistability) and *σ*(*t*) aids in accessing them by acting as a functional perturbation.

In the context of our toy example, the movement execution is accounted for by the functional mode, even though its initiation requires the involvement of the instantaneous signal *σ*(*t*). The phase flows used to that aim potentially involve a fixed point (i.e., mono-stable) or two fixed points (i.e., bi-stable) (the Excitator model can account for both cases [35]; Figures 2C,D). Both fixed point regimes are implemented via
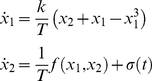
(2)where *x*_1,2_ are the state variables and *k*, *T* are constant. The function 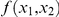 allows to manipulate the phase flow, where the mono-stable regime is realized for 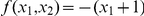 and the bi-stable regime for 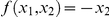. Both phase flows are characterized by a so-called separatrix, a structure in phase space that locally divides the flow in opposing directions. In these cases, movement execution requires that an (instantaneous) input *σ*(*t*) ‘kicks’ the system out of the fixed point and across the separatrix (see also [36], who report evidence for the existence of the corresponding threshold properties in humans, and [13]).

Consequently, the operational signal is responsible for the movement timing and initiation only—it does not dictate the system's dynamics. In contrast, the phase flows have to be complex enough to endow the functional modes with the existence of separatrices and (potentially) multistability, which is achieved via the introduction of nonlinearities. We thus expect that *Scenario 1* will be associated with a limited informational content of the operational signal and a high complexity of the functional modes.

#### Scenario 2

Here *τ_σ_*≈*τ_f_*, so that *σ*(*t*) acts on the phase flow on a time scale similar to the one of the functional mode, thus, constantly modifying its structure during the functional process. Consequently, the operational signal determines the functional dynamics by far.

An exemplar of such architectures is a dynamical formulation of equilibrium-point models that are well-known in the motor control literature [11], [37]–[40]. It consists of a single linear point attractor phase flow (for each effector),
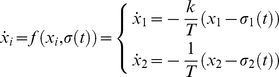
(3)where *x*_1,2_, *T* and *k* are as before. The operational signal *σ*_1,2_(*t*) determines the position of the linear point attractor in phase space—and thus the ensuing trajectory. Consequently, this scenario allows for the generation of trajectories of arbitrary complexity, but at the price of requiring the constant involvement of the operational signal *σ*_1,2_(*t*) that specifies the trajectories evolution. Indeed, as the operational signal largely prescribes the functional dynamics we expect its informational content to be high. The absence of nonlinearities, in contrast, is likely to result in a moderate phase flow complexity.

#### Scenario 3

Architectures in which *τ_σ_*≫*τ_f_* typically involve multiple functional modes since the slow change of *σ*(*t*) yields qualitative changes to the structure of the phase flow dynamics at critical points.

To obtain the required movement, we implement a van der Pol limit cycle (another instance of the generic Excitator model [35]; Figure 2B) as:
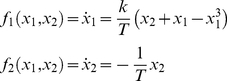
(4)(where *x*_1,2_, *T* and *k* are as before) and a linear point attractor, as these are the simplest systems describing rhythmic and discrete movements, respectively. In this implementation, *σ*(*t*) is responsible for sequencing phase flows; it sequentially selects a particular functional mode and can be considered approximately constant during the time the corresponding process evolves:
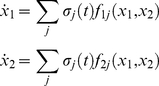
(5)where the operational signal 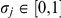 acts as an ‘on/off’ switch for each component functional mode so that only one mode is ‘on’ (i.e., activated) at each time step even though the selecting or switching parameter *σ_j_* is present throughout the entire movement sequence. This working of the selecting parameter *σ_j_* resembles the competition mechanisms in synergetics models of pattern recognition [41], [42].

While in contrast to *Scenario 2*, the operational signal in this scenario does not prescribe the functional dynamics, it is present to a far larger extent than in *Scenario 1*. Its informational content can thus be expected to lie in between that of the former two scenarios. The here combined use of linear and nonlinear phase flows predicts an intermediate functional mode complexity relative to *Scenario 1* (only nonlinear phase flows) and to *Scenario 2* (only linear phase flows).

#### Scenario 4

In the exemplar of these architectures, *σ*(*t*) is constant during the functional process (*τ_σ_*→∞), in other words, no operational signal is required (i.e., the system is autonomous), and thus no informational content can be associated with it. Furthermore, it will not come as a surprise that we expect this scenario to be associated with the highest functional mode complexity since it has to account for all the functional dynamics.

The current implementation consists of a single 4-dimensional phase flow exhibiting one attractor that controls the entire musical phrase as a whole. Such dynamics is achieved via an inhibitory coupling of simpler phase flows, like the ones described in *Scenario 3*:
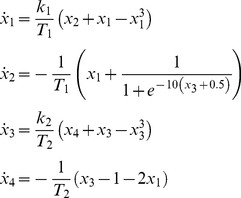
(6)where *x_1–4_*, *T_1,2_* and *k_1,2_* are as before. Alternative implementations could be achieved by coupling the simple phase flows through an additional slow varying state (or phase) variable [43], [44].

### Measures of Complexity

We quantify the degree of control required in the four scenarios, distinguishing between the control attributed to the operational signals or to the phase flow. For the former ones we calculate their Shannon entropy [45] (*H*), thus evaluating their informational content. As an additional measure to evaluate how much of the output trajectories dynamics is contained into *σ*(*t*) we also calculate their mutual maximum cross-correlation (*MCrC*).

For the phase flows, we quantify the complexity of the vector field. Given equation (1) for a phase flow and ignoring the effect of *σ*(*t*) uniformly sampling the phase space corresponds to obtaining a uniform distribution for the state variable **x** (in all that follows we adopt a vectorial representation for an *N*-dimensional system). As a consequence, its Shannon joint entropy maximally is *H*(**x**) = *H^max^*. The phase flow structure is quantified as *ΔH* = *H^max^*−*H*(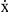). In other words, we compute the entropy reduction due to the application of the (vector field) function **f**(.) onto the random sample of the state variable **x**. (In the Supporting Information –file Text S1- we provide more technical details of all calculations involved as well as a brief additional discussion on their interpretation). Several other measures (such as singular value decomposition, entropy, joint entropy and mutual information) applied to time series generated by the phase flows utilized, have been tested in quantifying the functional modes' complexity, always giving converging results.

## Results

In the following we illustrate how the representative functional architectures generate the time course of our toy problem (see Figure 1).

In *Scenario 1* (see Figure 4 and Video S1 with Video Legends S1), the phase flows (monostable and bistable - fingers uncoupled) remain constant during the functional process. Three inputs (*τ_σ_*≪*τ_f_*) act upon the first finger's monostable phase flow (one per movement cycle) and two subsequent inputs upon the second finger's bistable phase flow. Notice that *σ*(*t*) operates upon the second and fourth dimensions of *x* that account for the velocities of the fingers' movements.

In *Scenario 2* (see Figure 5 and Video S2 with Video Legends S1), the phase flows (linear point attractors - fingers uncoupled) change at the same time scale as the functional process (*τ_σ_*≈*τ_f_*), since the position of the attracting fixed point is constantly assigned by the operational signal.

In *Scenario 3* (see Figure 6 and Video S3 with Video Legends S1), the phase flows (linear point attractors and limit cycle) change only at critical moments during the functional process due to the slow change of *σ*(*t*). In the initial period, both fingers are at rest. Then, the operational signal activates the functional mode of finger 1 (blue line in Figure 6) resulting in a finger oscillation. The functional modes are stable and constant during this period. Then the first mode is deactivated followed by the activation of the second mode (green line). Thus, *Scenario 3* is characterized by a consecutive activation of functional modes remaining active during a period substantially larger than the time scale of the relevant functional process (here the finger movement). As a consequence, the functional modes have to be substantially rich in complexity to account for the functional dynamics while operational signals have to be present throughout the process.

In *Scenario 4* (see Figure 7 and Video S4 with Video Legends S1), the 4-dimensional phase flow remains constant during the functional process since there is no operational signal involved. The whole function is accounted by the unique 4-dimensional complex attractor. Please note that in this scenario, the two finger movements are coupled by necessity, whereas in all previous scenarios this may or may not be the case.

**Figure 8:**
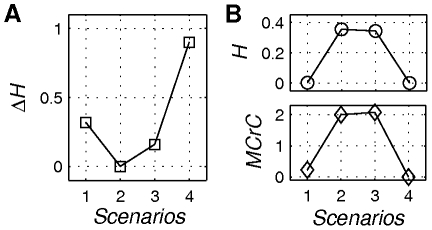
Complexity evaluation. Complexity of the operational signal (panel A) and the functional modes (panel B). The degree of control of the operational signal quantified via the Shannon entropy (*H*) and the maximum cross-correlation (*MCrC*) is high in *Scenarios 2* and *3*, to drop remarkably in *Scenario 1* and vanish in *Scenario 4*. The complexity of the functional modes *ΔH* is zero in *Scenario* 2, moderate in *Scenarios 1* and *3* and maximal in *Scenario 4*. Notice that *Scenario 1* qualifies as an efficient functional architecture, since it combines a minimal operational signal with a phase flow of moderate complexity.

Subsequently, the scenarios are evaluated via the application of complexity measures separately for the functional modes' phase flows and the operational signals involved. As can be appreciated from Figure 8, the measures confirmed the prediction of a ‘functional mode – operational signal complexity’ trade-off between *Scenarios 1*and *4* (with constant phase flows during the functional process) and *Scenario 2* (with flow changes at a similar time scale as the function) and *Scenario 3* (with very slow and intermittent time flow changes). In particular, both the operational signal's entropy and its cross-correlation with the system's output is zero in *Scenario 4* (*τ_σ_*→∞ - that is, *σ* is practically constant during the functional process), while being minimal in *Scenario 1* (*τ_σ_*≪*τ_f_*) and much larger in Scenario *2* & *3*. (One would also expect a bigger difference between *Scenarios 2* & *3*. However, the simplicity of our toy example does not allow this to become evident.) On the other hand, *ΔH* increased from zero in *Scenario 2* (*τ_σ_*≈*τ_f_*) to intermediate values for *Scenarios 1* & *3* (between which *Scenario 1* exhibits higher functional mode complexity) and finally, to a maximum value in *Scenario 4*.

*Scenario 1* qualifies as an efficient functional architecture, since it combines a minimal operational signal with a phase flow of moderate complexity. In other words, it appears to be computationally efficient to “precode” the functional dynamics to a certain degree with minimal intervention as the dynamics is executed.

## Discussion

We proposed a framework for functional architectures for the execution of complex functional processes. Accordingly, functional architectures contain two functionally distinct ingredients: a repertoire of functional modes, conceptualized as phase flows, and additional operational influences acting at a continuum of time scales relative to the ones pertaining to the functional modes. Below, we discuss the implications of our approach for functional modes, functional architectures, learning and related issues as well as neuro-scientific evidence in support of the representative architectures.

### Functional modes

By conceptualizing functional modes in terms of phase flows, our approach brings together the main features of functional modes in the context of motor control (i.e., motor primitives, synergies, and GMPs) as found in the dominating views in the literature. As the modes are defined through their topology, they combine class-invariant properties with within-class variation that allows for adjustments to specific task constraints.

In that sense the functional modes here proposed reveal a functional resemblance to generalized motor programs [9], [17]—though motivated from a diametrically opposing theoretical perspective. In addition, functional modes may be super-posed [10], [20] via the (slow) operational signal. In our architecture exemplified in *Scenario 3* the modes were super-positioned by setting one *σ* to one with the others vanishing. In principle, however, multiple *σ* may be competing and obtain any value (between zero and one) for an arbitrary duration of time, as for instance in perceptual categorization [2], [41], [42]. In fact, our approach and the modular primitives proposed by Musa-Ivaldi and colleagues are distinct in two major aspects. First, the primitives identified by Musa-Ivaldi *et al.* are defined in terms of hard-wired neural circuits in the spinal cords rather than in terms of (abstract) functional objects. The neurally-identified modules, however, are not at odds with our dynamically motivated architectures: it may well be that which neural modules are assembled and how they are super-positioned depends on the functional mode utilized in a given task context. Second, the domain of operation of Mussa-Ivaldi *et al.*'s spinal modules is limited to motor behaviors while our approach aims at a larger degree of generality including perceptual and cognitive processes. Finally, the executive stability requirement guarantees preservation of function in the face of perturbations. Several authors have previously proposed that fixed points and limit cycles constitute the building blocks of the (human) motor control system [13], [14], [22]. While (the flow pertaining to) these structures are included here, our general formulation allows (in principle) for numerous other flow topologies.

Regardless, some of the functional modes here utilized to illustrate the architectures have been identified as being used by humans (and other species) in the literature. In the motor control literature, rhythmic movements conceived of as limit cycles have been studied in-depth by various groups [46]–[49], which has shown that a diversity of nonlinear oscillator ingredients can be utilized so as to match particular task demands [34]. Similarly, discrete movements have been conceptualized as fixed points [14], [50] although they have been rarely explicitly identified (but see [13], [51], [52]). Moreover, which functional mode is used in (motor) precision aiming (a phase flow structured by a limit cycle or two fixed points) has recently been shown to be dependent on the task's difficulty (i.e., the degree of accuracy required relative to the target distance [52]). Changes of task difficulty within which a particular functional mode was utilized resulted in the structural task-dependent phase flow adjustments. All these instances, in other words, provide exemplars of class invariance as well as within class variability.

In the non-motor domain, distinct dynamical elements have been proposed to be associated with specific functional processes. For instance, an ‘s’ in a spoken word may be perceptually categorized as /s/ or /sh/, depending on the position of the tongue against the palette, and the transition from one category (or perceptual functional mode) to the next occurs abruptly (indicative of class invariance). At the same time, different speakers will pronounce an ‘s’ differently due to various (among others) anatomical differences of their articulator systems, indicating within class variability, but perceivers may still hear an /s/ [4]. In the visual domain, perceptual categorizing has also been mapped onto specific topological structures. For instance, honey bees [29] and humans [30] have been shown to be more sensitive to (static) topological properties of objects (such as connectivity, presence versus absence of holes) than to local features distinguishing the objects (but see [32], [33] for criticism). In a similar vein, changing topological relations in geographic events (e.g., the motion of a hurricane relative to a peninsula) are, next to non-topological features, used for their categorization [31], [53]. How such changes map onto phase flow patterns remains to be seen, however. Regardless, visual perception have been shown to exhibit characteristics pertaining to attractor dynamics (such as multistability and hysteresis, [54], [55]). The corresponding perceptual stability has been shown to depend on (biophysical) processes that stabilize the activation of individual neurons in ensembles of detectors and excitatory and inhibitory interactions among them [54]. In other words, while the question whether topologies represent visual primitives can presently neither be firmly confirmed nor refuted, there are strong indications that visual processing adheres to nonlinear dynamical principles, and thus lends itself naturally for an interpretation within the present framework.

### Functional architectures

We implemented specific realizations of the processes (as scenarios) for the prototypical architectures. Each scenario in principle allows for the implementation of infinitely many other flows (be it of the same or a higher dimension) than those here utilized. The corresponding topology, however, invariably grants the system with threshold-like properties (via the presence of a separatrix; as in *Scenario 1*); may reveal a dependency on a competition or switching parameter *σ* (as in *Scenario 3*). In other words, while quantitative aspects of the flows may vary, the additional (operational) influences that are required so as to perform a complex process, if any, is independent thereof for a given topology. That is, while the phase flow complexity may to a minor degree depend on the flow details, and, similarly, the *σ*s' entropy as well as its correlation with the system's output on task specifics, the degree of efficiency of the various scenarios will be largely independent thereof. We tested this argument computationally by creating large numbers of phase flows with varying topologies for a given scenario. In all instances the measures of complexity provided the same results.

Numerous examples of the architectures here illustrated can be found in the literature. For instance, equilibrium point control [11], [37]–[40] as well as Bullock *et al.*'s handwriting architecture [5] can be viewed as instantiations of *Scenario 2*. While in both cases the time scale of the operational signal *σ*(*t*) and the evolving trajectory may not match exactly (one issue pertaining to equilibrium point models is the speed at which the equilibrium point is set), under both models the system's evolution is largely determined by the repetitive resetting of the equilibrium point [37], [38] or GO signal in [5]. Examples of the slow operation of a competition or switching parameter *σ* relative to the functional modes' dynamics pervade the perceptual literature [2], [41], [42] and are likely to be involved in many cognitive processes. The existence of very brief impacts on flows (as in *Scenario 1*) is known in the motor control literature [13], [51], [52], where they are sometimes interpreted in terms of a ‘clock’ mechanisms [56], [57]. The simplest and minimal example of autonomous processes (*Scenario 4*) is purely oscillatory processes (i.e., processes governed by limit cycles), exemplars thereof abound in the motor control literature.

We outlined a framework for two-layered hierarchies; the number of layers, however, can easily be expanded (see also [2]), and will, in all likelihood, exceed two layers in complex processes, as for instance in language production and perception. Language is a hierarchical system containing multiple levels ranging from phonetics to meaning (of words, sentences, text, etc. [58], [59]). It is known that phonemes, the entities at the lower end of the linguistic hierarchy underlying communication, can be conceived of as dynamic meaningful categories [4]. Word recognition, a process which arguably takes place on one level above phonemes processing, has also been modeled in terms of attractor dynamics [60]. Both levels, as well as the higher ones, may very well be architecturally related as exemplified in *Scenario 3* (see also [2], [61]. Indeed, as functional modes are typically associated with elementary processes, it is likely that the more complex a process, the more layers are implicated.

Finally, we conceptualized functional modes and the additional effects thereon as operating independently. Obviously, this need not be the case; it may well be that, at least in some cases, both architecture ingredients are (uni- or bi-directionally) coupled. For instance, the instantaneous *σ*(*t*) pulses could well be constrained to occur with differential probability in different parts of phase space. This issue is, however, beyond the scope of the present study.

### Efficiency and Learning

The distinction between the dynamic repertoire and the operational signals allowed us to quantify the functional modes' complexity as well as the operational signals' entropy and correlation with the output. In other words, it allowed us to evaluate the different architectures' efficiency. Our results, in that regard, clearly showed a trade-off between the degree of (external) operation required and the complexity of the functional modes: the more complex the mode's topology the less the dependency on the operational signal. The mechanisms associated with *Scenario 1* came to the fore as most effective: the complexity of a functional mode's phase flow is moderate (at least relative to the extremes of Scenario 2 & 4) while requiring little external operation. The latter, in that regard, owes to the multistability (due to the presence of the separatrix) of the associated systems.

Similarly but inversely to their efficiency, the architectures' flexibility also goes approximately hand in hand with the operational effects required, at least, under the assumption that phase flow-governed movements cannot be (easily) stopped once initiated. Everyday experience is open to interpretation along these lines: who of us does not know the feeling of being unable to terminate an ongoing but erroneous movement? The price for the liberation from operational influences is paid in terms of a flexibility loss, while, inversely, flexibility comes at the costs of high external influences. Which architecture is best utilized will thus be context dependent: unpredictable contexts demand for high flexibility—do not even allow for complex functional modes, while the utilization of architectures whose functionality heavily depends on operational signals would be wasteful for the performance of repetitive and standardized actions.

The possibility of accomplishing a task via different architectures sheds a novel light on motor equivalence, that is, the phenomenon that task achievement can be achieved via various motor means [9]. For instance, writing can be performed with the right and left hand, as well as with a pen held between the lips or toes [9]. We demonstrate that task accomplishment, next to its realization via different end-effectors (in the context of motor behavior), can also be realized via distinct functional architectures.

Our approach opens up new avenues for the learning of function too. In dynamical approaches to motor behavior, perception and cognition, the learning of a particular task is generally viewed in terms of the creation of a new attractor and/or stabilization of an existing one [62] and corresponding dimensionality changes [63], [64], that is, as enduring changes at the level of existing functional modes. The present framework embraces these conceptions, but additionally predicts that skill acquisition may be extended to the learning of novel functional modes as well as architectures. An important question, in that regard, is whether the learning of multiple architectures using particular functional modes would transfer to, or at least facilitate learning a task in which different modes need to be used.

The learning of novel functional modes can, tentatively, be linked to a particular instance of learning referred to as ‘chunking’. Chunking is a process commonly believed to play a role in perceptual, motor, and cognitive sequence learning during which multiple (functional) elements are integrated into one larger ‘chunk’ [65]–[68]. While chunking is generally believed to be a process in which the smaller units are somehow linked together, it may well be that the newly formed chunks are fundamentally (topologically) different from the basic units. That is, while in the former case the individual units (modes) remain traceable; in the latter case the process underlying the function fundamentally changes (cf. [61]). From our perspective, simply linking basic units will hardly, if at all, diminish the control (or otherwise processing) demands, which requires a fundamentally different chunk organization. In line with this intuition, recent work in artificial intelligence suggests that ‘horizontal learning’ (re-using existing capabilities at every learning step) is associated with computationally less efficient than ‘vertical learning’ (in which new capabilities are created; [6]. In that regard, the process of reorganizing the architecture underlying a particular skill may well provide a new complementary window into the automatization of motor behavior during learning [9], [69].

### Neural support for the representative architectures

The functional architectures here outlined are per definition abstract, that is, one may well ask if their existence is supported by neuro-scientific data. As for the functional modes, it has recently been formally shown that networks composed of firing rate neurons are able to generate phase flows with distinct topologies [70]. Similarly, networks of spiking neurons [71] are able to generate so-called heteroclinic cycles (i.e., low dimensional orbits in higher dimensional space; see also [72]). In biological systems, it is well known that populations of neurons may be active in a coordinate fashion, which effectively reduces the population's dynamics. Significantly, the dynamics of neurons constituting central pattern generators are typically constrained so as to produce a limit cycle dynamics, which is reflected in the ensemble dynamics phase space [73]. In other words, dynamical models as well as biological data indicate that the ensemble dynamics of populations of neurons may effectively reduce to a structured flow in phase space, that is, a functional mode.

The time scale separation as exemplified in *Scenario 3* resembles the one as reported by Kiebel *et al.* [2]. These authors argued that the time scale separation found in environmental events is reflected in the hierarchical organization of the nervous system, in particular the cortex. Structurally, the hierarchy is formed via convergence and divergence of forward and backward connections, while their differential functionality introduces a temporal (and spatial) separation of scales of operation. Presumably, processes in the primary areas occur faster than the modular influences thereon from the higher levels. Assuming that the functional modes are generated in the primary (and/or secondary) areas, Kiebel *et al.* arguments may well be consistent with the (closely-associated) architecture here exemplified in *Scenario 3*.

Evidence favoring biological realism for the different architectures has been found (or suggested) in various contexts. For instance, the operational signals *σ*(*t*) pertaining to *Scenario 2*-like architectures can be associated with equilibrium points in the corresponding models [11], [37]–[40], which are inherently physiologically motivated. In these models, a limb's equilibrium position is defined by the flexor and extensor's length-tension functions, and movements are made by shifting the length-tension functions (see [11] for an extension beyond paired agonist-antagonist interpretations). The adjustments of the length-tension curves are brought about via the *α*-(motor) neurons solely (the *α*-model [37], [38] or in conjunction with *γ*-system and muscle spindle feedback (the *λ*-model [74]–[76]).

The instantaneous signals *σ*(*t*) have previously been associated with timing mechanisms (‘clocks’) [13], [51]. In fact, the notion of brief pulses initiating timed movements is well established in the psychological literature [56], [57], and is accompanied by a plentitude of neuro-imaging studies aiming to identify the timing mechanism's anatomical substrate (for a review, see [77]). The cerebellum [78]–[80], and basal ganglia [77] have been forwarded as candidate structures, in that regard. This brief overview readily indicates that there are multiple indications in favor of both the neural generation of functional modes as well as the existence of the various operational signals in the nervous system.

### Conclusion

We outlined a general framework for functional architectures controlling complex behavioral processes that contains two functionally distinct elements that (potentially) operate on different time scales. Our analysis offers a theoretical justification for the fact that complex behavioral processes are composed of functional subunits. This conceptualization opens a new theoretical window into the control of complex processes, learning and automatization as well as chunking. From a more applied perspective, our insights may have offshoots to robotics and related fields (where some of the here utilized primitives are already implemented [14], [24]–[27]), learning and rehabilitation.

## Supporting Information

Text S1 **Supplementary information on model formulation, simulations and complexity measures.**
(DOC)

Video S1 **Simulation of** ***Scenario 1*** **.**
(MP4)

Video S2 **Simulation of** ***Scenario 2.***
(MP4)

Video S3 **Simulation of** ***Scenario 3*** **.**
(MP4)

Video S4 **Simulation of** ***Scenario 4*** **.**
(MP4)

Video Legends S1 **Legends of videos.**
(DOC)
