# Supplementary material for: Complex Processes from Dynamical Architectures with Time-Scale Hierarchy
Source: PLoS One. 2011 Feb 10;6(2):e16589. doi: 10.1371/journal.pone.0016589 (PMC3037373; doi:10.1371/journal.pone.0016589)
Supplement: Text S1 — Supplementary information on model formulation, simulations and complexity measures. (DOC) [file pone.0016589.s001.doc]

1. **Simulations**

All simulations were carried out in MATLAB. For the integration of the dynamical systems, a Runge-Kutta algorithm of 4^th^ order has been used.

1. **Systems’ equations**

In the following, we present in detail the equations of the systems exemplifying the four *Scenarios* of the proposed architecture. In all cases, $\mathbf{x}=\left(x_{1},x_{2},x_{3},x_{4}\right)\in$ℝ^4^ are the state variables of the system (where $\left(x_{1},x_{2}\right)$ and $\left(x_{3},x_{4}\right)$ refer to the first and second effector respectively), $T_{1,2}\in\left(0,+\infty\right)$ are the effectors’ main time scale parameters while $k_{1,2}\in\left(0,+\infty\right)$ introduce a time scale separation between the state variables of each effector’s phase flow.

*Scenario 1*

where $T_{1}=10$, $T_{2}=1$, $k_{1}=100$ and $k_{2}=10$. $\sigma_{2,4}\left(t\right)$ is the operational signal (instantaneous input).

*Scenario 2*

where $T_{1}=T_{2}=1$ and $k_{1}=k_{2}=10$. $\sigma_{1-4}\left(t\right)$ is the operational signal acting as a multidimensional equilibrium point control parameter.

*Scenario 3*

where

$$\begin{matrix} f_{11}\left(x_{1},x_{2}\right)=-\frac{k_{11}}{T_{11}}\left(x_{1}+1\right) \\ f_{12}\left(x_{1},x_{2}\right)=\frac{k_{12}}{T_{12}}\left(x_{2}+x_{1}-x_{1}^{3}\right) \\ f_{12}\left(x_{1},x_{2}\right)=-\frac{1}{T_{11}}x_{2} \\ f_{22}\left(x_{1},x_{2}\right)=-\frac{1}{T_{12}}x_{2} \\ f_{31}\left(x_{3},x_{4}\right)=-\frac{k_{21}}{T_{21}}\left(x_{3}+1\right) \\ f_{32}\left(x_{3},x_{4}\right)=-\frac{k_{22}}{T_{22}}\left(x_{3}-1\right) \\ f_{41}\left(x_{3},x_{4}\right)=-\frac{1}{T_{21}}x_{4} \\ f_{42}\left(x_{3},x_{4}\right)=-\frac{1}{T_{32}}x_{4} \end{matrix}$$

and $T_{11}=T_{21}=T_{22}=1$, $T_{12}=4$, $k_{11}=k_{12}=k_{21}=k_{22}=10$. $\sigma_{i,j}\left(t\right)$ is the operational signal (selecting or switching parameters), where indexes $i,j=1,2$ run across effectors and phase flows, respectively.

*Scenario 4*

where $T_{1}=8$, $T_{2}=86$, $k_{1}=100$ and $k_{2}=50$.

1. **Operational signals**

No claim for the generating mechanisms of the operational signals is made in the present work. The ones used in the simulations where chosen such as that the resulting multidimensional operational signals are non-autonomous and their different dimensions are uncorrelated.

The instantaneous input $\sigma\left(t\right)$ of *Scenario 1* is generated as rectangular pulses of a very short duration in comparison with their amplitude, thus approximating the *δ* impulse function.

The equilibrium point parameters of *Scenario 2* are generated through fast linear differential equations driven by the target time series:

,

where $T_{\sigma}=10$ and $x_{1,2}^{*}$ are the position target time series of the movement plotted in Figure 1 of the main text.

The selection parameters of *Scenario 3* are generated similarly:

where $T_{\sigma}=10$ and $x_{1,2}^{*}$ are as before, and $\sigma_{11}+\sigma_{12}=1$ and $\sigma_{21}+\sigma_{22}=1$ at every time step.

1. **Quantification**

Entropy or (joint entropy for multi-dimensional data) were calculated following the Shannon entropy definition [1]:

$H=\frac{-\sum_{i=1}^{n_{bins}^{N}} p_{i}\ln p_{i}}{\ln n_{bins}}$,

where the probability distribution $p_{i}$ of a data series of dimension *N* is calculated through the construction of an *N* dimensional histogram with $n_{bins}$ bins per dimension. The normalization with $\ln n_{bins}$ constrains the values of *H* in the range$\left[0,N\right]$.

1. **Operational signals**

Given all the above, the calculation of the operational signal entropy for each scenario is given by:

$\begin{matrix} H_{Sc1}=H\left(\sigma_{2}\right)+H\left(\sigma_{4}\right) \\ H_{Sc2}=\sum_{i=1}^{4} H\left(\sigma_{i}\right) \\ H_{Sc3}=H\left(\sigma_{1}\right)+H\left(\sigma_{2}\right) \end{matrix}$ .

The calculation of the maximum cross correlation between the output of the constructed systems and the operational signals is given by:

$\begin{matrix} MCrC_{Sc1}=corr\left(x_{2},\sigma_{2}\right)+corr\left(x_{4},\sigma_{4}\right) \\ MCrC_{Sc2}=\sum_{i=1}^{4} \left(corr\left(x_{i},\sigma_{i}\right)\right) \\ MCrC_{Sc3}=corr\left(x_{1},\sigma_{1}\right)+corr\left(x_{2},\sigma_{1}\right)+corr\left(x_{3},\sigma_{2}\right)+corr\left(x_{4},\sigma_{2}\right) \end{matrix}$.

In all cases *corr*(.) is the cross correlation function given by:

$corr\left(a,b\right)=\frac{E\left(ab\right)-E\left(a\right)E\left(b\right)}{\sqrt{E\left(a^{2}\right)-E^{2}\left(a\right)}\sqrt{E\left(b^{2}\right)-E^{2}\left(b\right)}}$,

where $E\left(.\right)$ is the mean. The lag between *a* and *b* data series chosen is the one that maximizes the above quantity.

1. **Phase flows**

For the calculation of the ∆*H* phase flow measure we consider a subset of the phase space relevant to the task, common to all phase flows used: a hypercube of side spanning real numbers in the range [-2 2]. If we sample the state variable $\mathbf{x}=\left(x_{1},x_{2},x_{3},x_{4}\right)\in$[-2 2]^4^ uniformly, we get a data set $\mathbf{X}=\left(X_{1},X_{2},X_{3},X_{4}\right)\in$[-2 2]^4^ with maximal joint entropy $H\left(\mathbf{X}\right)=H^{\max}=N$ (where *N*=4). Then, we calculate the respective data set from the equation (where **f**(.) is the multidimensional functional form of the phase flow), which in turn has joint entropy . From there, it follows: . Given that the joint entropy of uncorrelated variables equals the sum of entropies of each variable, the specific calculation for each scenario is described by the equations:

(Recall that for *Scenario 1*, we considered two 2-dimensional phase flows - a monostable and a bistable one [2]. With regards to *Scenario 3* we have considered a limit cycle (2-dimensional) and two linear 1-dimensional phase flows.)

We consider the reduction ∆*H* of entropy due to the process **f**(.) on the random data set $\mathbf{X}$ as an evaluation of the structure of the phase flow (described by **f**(.)). One should notice that the minimum value ∆*H=0* corresponds to the linear phase flow (where **τ** is a constant diagonal matrix) since in this case, if $\mathbf{X}$ has a uniform distribution, will also have one. This fact can be viewed as an evidence for ∆*H* scaling in general with the deviation from the linear system.

**References**

1. Weaver W, Shannon CE (1963) The mathematical theory of communication: University of Illinois Press Urbana.

2. Jirsa V, Scott Kelso J (2005) The excitator as a minimal model for the coordination dynamics of discrete and rhythmic movement generation. Journal of motor behavior 37: 35-51.

Video captions:

**Video S1**: *Scenario 1* is illustrated by the vector fields of the phase flows (monostable and bistable) together with the output trajectories (top panel) as well as by the output time series (positions *x*_1,3_ and operational signals *σ*_2,4_(*t*) - bottom panel) as they evolve in time. Blue and green discriminate between the first and second finger, respectively; a small black filled circle denotes an attracting fixed point, while a non-filled circle shows the current state of the system is in the phase space. The phase flows remain constant during the functional process (*τ_σ_*≪*τ_f_*), while the operational “kicks” initiate one movement cycle per stimulus for the monostable flow (finger 1, top left panel) and one half cycle per stimulus for the bistable phase flow (finger 2, top right panel).

**Video S2**: *Scenario 2* is illustrated by the vector fields of the phase flows (linear point attractors) together with the output trajectories (top panel) as well as by the output time series (positions *x*_1,3_ and operational signals *σ*_1,3_(*t*) - bottom panel) as they evolve in time. (For symbols and colour coding, see Video S1.) The phase flows change at the same time scale as the functional process (*τ_σ_*≈*τ_f_*), since the position of the attracting equilibrium point is constantly assigned by the operational signal. The continuous evolution of the vector fields’ structure during the functional process can be easily observed: first the phase flow corresponding to finger 1 (top left panel) is modified since the point attractor moves first from position *x*_1_=-1 (resting) to position *x*_1_=1 (key pressing), and after a while it returns back under the driving of the operational signal *σ*_1_(*t*) (as always). This ‘event’ is repeated three times, once for every movement cycle. Subsequently, the same happens for finger 2 (top right panel) under the driving of *σ*_3_(*t*). Notice that there is a small time lag between *σ*_1,3_(*t*) and *x*_1,3_, respectively, that depends on their relative time scales. (The video is slowed down by a factor of 20 for clarity when the operational signal varies.)

**Video S3**: *Scenario 3* is illustrated by the vector fields of the phase flows (top panel) as well as by the output time series (positions *x*_1,3_ and operational signals *σ*_1,2_(*t*) - bottom panel) as they evolve in time. (For symbols and colour coding, see Video S1.) The phase flows only change at brief moments during the functional process due to the slowly changing operational signal. At first, both fingers are at rest since the respective active phase flows are characterized by a single point attractor at the resting position *x*_1,3_=-1. Then, *σ*_1_(*t*) changes from -1 to 1 and, as a result, a limit cycle phase flow is activated for finger 1 (top left panel), which starts to oscillate. After three movement cycles *σ*_1_(*t*) becomes -1 (again) and the limit cycle is deactivated and replaced by the (initial) point attractor phase flow. As a consequence, finger 1 returns back to the resting position. Then, a similar process occurs for finger 2 where two different point attractor phase flows (with point attractors at the resting and the “key pressing” position respectively) alternate as the operational signal *σ*_2_(*t*) is modified from -1 to 1 and backwards. Notice that *σ*_1,2_(*t*) remains constant for long time periods relatively to the functional process (*τ_σ_*≫*τ_f_*). (The video is slowed down by a factor of 20 for clarity when the operational signal varies.)

**Video S4**: Scenario 4 is illustrated by the output trajectory in the phase space (two different 3-dimensional projections – left and right top panel) as well as by the output time series (positions *x*_1,3_ and operational signal *σ*(*t*) – bottom panel). Blue and green discriminate between first and second finger (coupled) only for the time series plot. The phase flow remains constant during the functional process since there is no operational signal involved. Although these are just 3-dimensional projections of the phase flow, one can observe the spiral of three movement cycles of finger 1 on the plane *x*_1_-*x*_2_ (top left panel), followed by a slower one of finger 2 on the plane *x*_3_-*x*_4_ (top right panel).
