# Supplementary material for: Complex Processes from Dynamical Architectures with Time-Scale Hierarchy
Source: PLoS One. 2011 Feb 10;6(2):e16589. doi: 10.1371/journal.pone.0016589 (PMC3037373; doi:10.1371/journal.pone.0016589)
Supplement: Video Legends S1 — Legends of videos. (DOC) [file pone.0016589.s006.doc]

**Video legends**

**Video S1. Simulation of *Scenario 1*.** *Scenario 1* is demonstrated by the vector fields of the phase flows (monostable and bistable) together with the output trajectories (top panel) as well as by the output time series (positions *x*_1,3_ and operational signals *σ*_2,4_(*t*) - bottom panel) as they evolve in time. Blue and green discriminate between the first and second finger, respectively; a small black filled circle denotes an attracting fixed point, while a non-filled circle shows the current state of the system is in the phase space. The phase flows remain constant during the functional process (*τ_σ_*≪*τ_f_*), while the operational “kicks” initiate one movement cycle per stimulus for the monostable flow (finger 1, top left panel) and one half cycle per stimulus for the bistable phase flow (finger 2, top right panel).

**Video S2. Simulation of *Scenario 2.*** *Scenario 2* is demonstrated by the vector fields of the phase flows (linear point attractors) together with the output trajectories (top panel) as well as by the output time series (positions *x*_1,3_ and operational signals *σ*_1,3_(*t*) - bottom panel) as they evolve in time. (For symbols and colour coding, see Video S1.) The phase flows change at the same time scale as the functional process (*τ_σ_*≈*τ_f_*), since the position of the attracting equilibrium point is constantly assigned by the operational signal. The continuous evolution of the vector fields’ structure during the functional process can be easily observed: first the phase flow corresponding to finger 1 (top left panel) is modified since the point attractor moves first from position *x*_1_=-1 (resting) to position *x*_1_=1 (key pressing), and after a while it returns back under the driving of the operational signal *σ*_1_(*t*) (as always). This ‘event’ is repeated three times, once for every movement cycle. Subsequently, the same happens for finger 2 (top right panel) under the driving of *σ*_3_(*t*). Notice that there is a small time lag between *σ*_1,3_(*t*) and *x*_1,3_, respectively, that depends on their relative time scales. (The video is slowed down by a factor of 20 for clarity when the operational signal varies.)

**Video S3. Simulation of *Scenario 3*.** *Scenario 3* is demonstrated by the vector fields of the phase flows (top panel) as well as by the output time series (positions *x*_1,3_ and operational signals *σ*_1,2_(*t*) - bottom panel) as they evolve in time. (For symbols and colour coding, see Video S1.) The phase flows only change at brief moments during the functional process due to the slowly changing operational signal. At first, both fingers are at rest since the respective active phase flows are characterized by a single point attractor at the resting position *x*_1,3_=-1. Then, *σ*_1_(*t*) changes from -1 to 1 and, as a result, a limit cycle phase flow is activated for finger 1 (top left panel), which starts to oscillate. After three movement cycles *σ*_1_(*t*) becomes -1 (again) and the limit cycle is deactivated and replaced by the (initial) point attractor phase flow. As a consequence, finger 1 returns back to the resting position. Then, a similar process occurs for finger 2 where two different point attractor phase flows (with point attractors at the resting and the “key pressing” position respectively) alternate as the operational signal *σ*_2_(*t*) is modified from -1 to 1 and backwards. Notice that *σ*_1,2_(*t*) remains constant for long time periods relatively to the functional process (*τ_σ_*≫*τ_f_*). (The video is slowed down by a factor of 20 for clarity when the operational signal varies.)

**Video S4. Simulation of *Scenario 4*.** Scenario 4 is demonstrated by the output trajectory in the phase space (two different 3-dimensional projections – left and right top panel) as well as by the output time series (positions *x*_1,3_ and operational signal *σ*(*t*) – bottom panel). Blue and green discriminate between first and second finger (coupled) only for the time series plot. The phase flow remains constant during the functional process since there is no operational signal involved. Although these are just 3-dimensional projections of the phase flow, one can observe the spiral of three movement cycles of finger 1 on the plane *x*_1_-*x*_2_ (top left panel), followed by a slower one of finger 2 on the plane *x*_3_-*x*_4_ (top right panel).
